# Multi-Hazard-Resistant Behavior of CFRP- and Polyurea-Retrofitted Reinforced Concrete Two-Column Piers under Combined Collision–Blast Loading

**DOI:** 10.3390/ma16103784

**Published:** 2023-05-17

**Authors:** Chen Fang, Tewodros Y. Yosef, Daniel G. Linzell

**Affiliations:** 1 Midwest Roadside Safety Facility, Department of Civil and Environmental Engineering, University of Nebraska-Lincoln, Lincoln, NE 68583, USA; cfang5@unl.edu; 2 Department of Civil and Environmental Engineering, University of Nebraska-Lincoln, Lincoln, NE 68586, USA

**Keywords:** multi-hazard resistance, CFRP retrofitting, polyurea coating, reinforced concrete piers, collision–blast loading, finite element analysis

## Abstract

This study investigated the multi-hazard resistance of highway bridge piers retrofitted with carbon-fiber-reinforced polymer (CFRP) and polyurea coating against the combined collision–blast loads and evaluated their effectiveness. Detailed finite element models of CFRP- and polyurea-retrofitted dual-column piers that considered the blast-wave–structure interactions and the soil pile dynamics were developed using LS-DYNA to simulate the combined effects of a medium-size truck collision and close-in blast. Numerical simulations were conducted to examine the dynamic response of bare and retrofitted piers under different levels of demands. The numerical results indicated that using CFRP wrapping or polyurea coating effectively mitigated the combined collision and blast effects and increased the pier’s resistance. Parametric studies were performed to identify an in situ retrofit scheme to control the parameters and determine the optimal schemes for the dual-column piers. For the parameters that were studied, the results showed that retrofitting at half the height of both columns at the base was identified as an optimal scheme to improve the multi-hazard resistance of the bridge pier.

## 1. Introduction

Highway bridges can be subjected to extreme hazards during their service life, such as earthquakes, vehicle collisions, fires, blasts, and landslides, in addition to supporting their self-weight and vehicle loads. Reinforced concrete (RC) multi-column piers are commonly used highway bridge support units. For piers that are located adjacent to travel lanes, if protective systems are not in place, they can be susceptible to collisions from moving vehicles. When an intentional or unintentional blast happens in association with the collision event, severe damage can occur to supporting piers, possibly causing critical service interruptions and potential collapse. Vehicle collisions and air blasts are considered extreme situations when designing a highway bridge [1]. However, these extreme events happened in Nashville, Tennessee [2]. A highway bridge over I-25 was exposed to a tractor-trailer truck collision with a subsequent blast, significantly damaging the columns and girders. The extreme demands from the multiple hazards are not explicitly considered in the current American Association of State Highway and Transportation Officials (AASTHOs) design codes [1], which utilize an equivalent static force in an attempt to replicate collision effects. As a result, exploration of their effects and study of potential quickly implemented, low-cost, in situ retrofit schemes could be beneficial for the state of practice.

Protective devices are often placed adjacent to bridge substructure units to prevent direct collisions. However, supporting elements are often located in a fashion where it is not feasible to economically place protective devices [3]. The use of innovative, efficient, and cost-effective retrofit schemes, such as the placement of protective “skins” made of carbon-fiber-reinforced polymers (CFRPs) and polymeric coatings, could have beneficial results. Attaching a CFRP to component surfaces was shown to enhance structural performance [4] by improving member capacities and ductility. CFRP composites are commonly bonded on the tensile face of concrete flexural elements to improve their bending resistance [5]. CFRP sheets are typically wrapped around compression members to confine the concrete, thereby increasing the axial capacity.

A few research studies have been conducted to evaluate the performance of RC components strengthened with CFRP under either impact or blast loading. Sha et al. [6] performed experimental and numerical studies that examined a CFRP-strengthened RC pier subjected to barge impacts. The results showed using the CFRP effectively reduced pier damage and improved pier impact resistance. Xu et al. [7] completed an experimental investigation on the impact behavior of cantilever CFRP-strengthened RC columns and showed that the lateral impact resistance could be improved. Swesi et al. [8] experimentally investigated the performance of CFRP-strengthened RC columns subjected to impact loads and showed positive effects when the number of CFRP layers increased. Liu et al. [9] conducted experimental and numerical studies on the impact behaviors of CFRP-strengthened circular RC columns and performed parametric studies to assess the effects of structural and load parameters on column performance. Hao and Tang [8] conducted numerical simulations of a cable-stayed bridge subjected to blast loads and investigated the effectiveness of the CFRP in enhancing bridge blast resistance. Jahami et al. [10] numerically evaluated the blast-loaded behavior of CFRP-strengthened RC beams and showed that CFRP improved the blast energy absorption. Yan et al. [11] experimentally and numerically examined the dynamic behavior of CFRP-strengthened RC columns subjected to close-in blast loading and evaluated the effects of critical parameters on the column blast performance. Hu et al. [12] conducted close-in blast tests on CFRP-retrofitted RC columns and investigated their residual capacities when the axial load ratio, CFRP thickness, longitudinal reinforcement ratio, and stirrup ratio varied. Several research studies also pointed out the limitations of using FRP, such as the high potential material cost and the durability of bonding epoxy [10,11].

Polyurea, which is created from a chemical reaction product of isocyanate and amine, offers potential performance advantages, such as better adhesion and lower material and labor costs. Limited studies have evaluated polyurea performance when placed onto steel or concrete substrates under impact or blast loading. Chen et al. [13] completed experimental and numerical studies that examined polyurea’s influence on the behavior of steel plates under impact loading. Raman et al. [14] experimentally and numerically examined the blast load response of a polyurea-coated RC slab. The results showed that the polyurea coating mitigated the slab damage and improved the capacity. Iqbal et al. [15] conducted an experimental investigation on the effect of polyurea coating on the survivability of concrete under blast loading and demonstrated its feasibility to enhance concrete blast resistance and improve concrete performance. Polyurea is a promising material for engineering practices to protect the bridge and bridge components against extreme loads.

Limited research has examined the performance of RC bridge piers subjected to collision and blast when supporting piers are strengthened using CFRP or polyurea. A few studies on FRP-wrapped or polyurea-coated RC components had limited scopes, focusing on slabs and beams [16,17,18]. This study aimed to evaluate the performance of dual-column piers strengthened using either CFRP wrap or polyurea coating when subjected to the combined collision–blast loads and examine the effectiveness of selected retrofit schemes in enhancing the pier’s resistance. Numerical models of the bare and strengthened piers were developed using LS-DYNA to compare the pier’s response to various extreme demands. Additionally, parameter studies were conducted to evaluate the effects of design parameters on the pier’s performance and the retrofit efficiency in mitigating the combined effects of a truck collision and air blast.

## 2. Numerical Modeling

Three-dimensional, fully nonlinear, finite element models of representative highway bridge piers strengthened using CFRP wrap and polyurea in situ retrofit schemes were developed using LS-DYNA. The models consisted of dual-column piers, their foundation systems, studied strengthening schemes (i.e., CFRP wrap and polyurea coating), a surrounding soil domain, and an air volume.

### 2.1. Element Formulation

Concrete for the pier columns, footings, piles, and caps was modeled using an 8-node, single-point integration, constant stress, and solid element. The reinforcement was modeled using a 2-node, Hughes–Liu beam element with a cross-section integration formulation. The CFRP and polyurea were simulated using a 4-node shell element with the Belytchko–Tsay formulation. To minimize the hourglass effect, Flanagan–Belytschko stiffness-based hourglass control was employed for the concrete, CFRP, and polyurea with a coefficient of 0.05 [19]. The surrounding soil and air were modeled using single-point multi-material arbitrary Lagrangian–Eulerian (MM-ALE) solid elements. Viscous hourglass control was used to model air with a coefficient of 1 × 10^−6^ [20].

### 2.2. Material Models

#### 2.2.1. Concrete

The *MAT CSCM Concrete model* was used to simulate the dynamic response of concrete for the dual-column pier. This concrete model was a continuous surface cap model that considered the concrete’s stiffness degradation, damage-based softening, and strain rate effect. Previous studies demonstrated the capability of the CSCM concrete model to predict concrete behavior when subjected to impact or blast loads [21,22,23]. The concrete damage in which concrete cracking or crushing occurred was quantified using a scalar damage parameter in this model. The damage parameter had a range between 0 (i.e., no damage) and 1 (i.e., total loss of concrete stiffness and strength). Strain rate effects were incorporated in the concrete model for the fracture energy, plasticity surface, and damage surface based on a viscoplastic formulation. The CSCM concrete model provides a parameter generation function based on the compressive strength and maximum aggregate size when the detailed material date is not available. Table 1 lists the concrete parameters for this study. An erosion algorithm in this model was also utilized to remove the highly strained elements of the deformed concrete, and the coefficient was conservatively set to 1.1 [24,25].

#### 2.2.2. Steel 

Steel reinforcement was explicitly modeled using LS-DYNA’s *MAT Piecewise Linear Plasticity* model. This model is an elastoplastic material model that incorporates yielding, plastic-strain-based failure, hardening, and strain rate effects. Table 1 lists the steel properties used in this study. Strain rate effects on steel strength were represented using the Cowper and Symonds model. In this study, the Cowper and Symonds coefficients were set to *C_s_* = 40 and *p_s_* = 5 [22]. Constraint-based contact was utilized to couple the reinforcement to its surrounding concrete using a *Constrained Lagrange in Solid* algorithm [19,22].

#### 2.2.3. Air and Blast

The *MAT Null* material model and a linear polynomial equation of state (EOS) were used to represent the air volume [19]. A TNT explosive was selected for this study and modeled using the LS-DYNA *MAT High Explosive Burn* model and the Jones–Wilkins–Lee (JWL) EOS. The JWL EOS simulates the blast pressure as a function of the relative volume of the explosive. A sphere explosive was contained in the air mesh by defining an initial fraction using the *Initial Volume Fraction Geometry* formulation.

#### 2.2.4. Soil 

The *MAT FHWA Soil* model was employed to simulate the soil behavior under impact or blast loading. This soil model can simulate strain softening, strain rate effects, kinetic hardening, and low confinement effects for the soil. This model was developed by the Federal Highway Administration (FHWA) to investigate the soil’s response when subjected to vehicle impacts [26]. Studies indicated that the soil model could accurately predict the impact or blast-loaded response of the soil [27,28]. Table 2 lists the soil material parameters.

#### 2.2.5. CFRP

The *MAT Enhanced Composite Damage* model was utilized to simulate the CFRP wrap with element properties modeled using the *Part Composite* formulation. This material model accounts for post-stress degradation and failure in tension, compression, and shear based on the Chang–Chang criteria [19]. This model can predict different failure modes of CFRP, including compressive fiber failure, compressive matrix failure, tensile fiber failure, and tensile matrix failure [19]. As indicated in the previous studies [5,6], the CFRP behavior is insignificantly affected by the strain rate, and thus, the strain rate effects were not considered in this study. Table 3 lists the CFRP material parameters for this study.

#### 2.2.6. Polyurea

**Figure 1:**
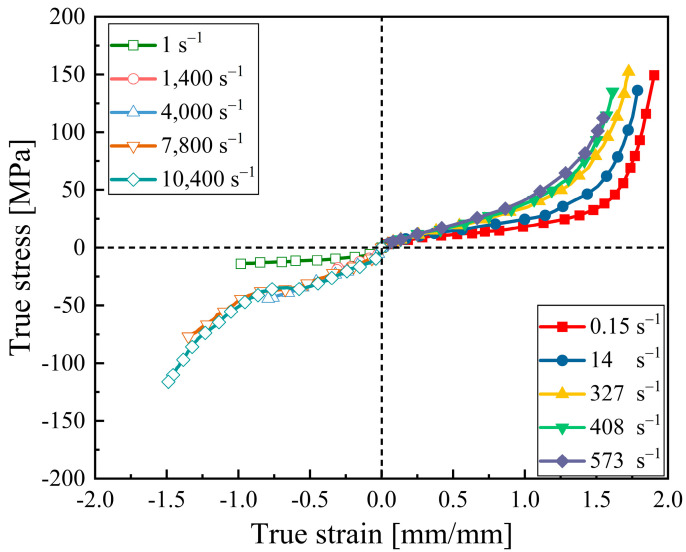
Stress–strain constitutive relations for polyurea (data obtained from [29]).

The *MAT Modified Piecewise Linear Plasticity* model was used to simulate the dynamic response of polyurea coating. It is an elastoplastic material that can incorporate strain-hardening, enhanced failure criteria, and strain rate effects using rate-dependent stress–strain curves. Polyurea rate-dependent properties were obtained from experimental tests provided by Roland et al. [29], as shown in Figure 1. In this study, the polyurea elastic modulus was set to 2520 MPa and the yield strength was 10 MPa. The Poisson’s ratio was set to 0.465 due to this being an almost incompressible material [13].

### 2.3. Contact Modeling

The CFRP was bonded to the pier column surfaces by explicitly modeling the epoxy adhesive. The *Automatic Surface-to-Surface Tiebreak* algorithm was used to model the adhesive contact and simulate FRP delamination. Contact failure was assessed using an interaction equation involving epoxy shear and tensile strengths, as expressed in Equation (1):

(1) $${\left(\frac{\left|\sigma_{n}\right|}{NFLS}\right)}^{2}+{\left(\frac{\left|\sigma_{s}\right|}{SFLS}\right)}^{2}\geq 1$$

where *σ_n_* and *σ_s_* are the interface normal and shear stresses, and *NFLS* and *SFLS* are the failure tensile and shear stresses. The epoxy properties were obtained from published studies [5,6], and the tensile and shear failure stresses were 32 MPa and 29.4 MPa, respectively. Polyurea is commonly sprayed onto concrete surfaces; as a result, the contact between the polyurea and concrete was also modeled using the *Automatic Surface-to-Surface Tiebreak* algorithm. Polyurea’s tensile and shear failure stresses were set to 1.04 MPa and 6.90 MPa, respectively, based on previous studies [30].

The Ford F800 single-unit truck (SUT) model with a total mass of 8000 kg impacted the dual-column piers along the longitudinal axis at specified velocities. Segment-based, penalty-type contact was used to model the interaction between the SUT and the pier using the *Contact Automatic Surface-to-Surface* formulation, and the static and dynamic friction coefficients were set to 0.3 [24,31]. The air blast was simulated from the TNT detonation and blast wave in the air using MM-ALE formulation in LS-DYNA. The blast wave and the pier were coupled through a penalty-based coupling algorithm using the *Constrained Lagrange in Solid* formulation. Interaction between the soil and the foundation system was also modeled using the *Constrained Lagrange in Solid* formulation with a friction coefficient of 0.315. Soil and air volumes were simulated as being infinitely continuous by specifying the *Boundary Non-Reflecting* algorithm in the exterior sides of soil and air domains, avoiding the effects of dynamic wave reflection.

### 2.4. Validations of the Numerical Modeling Approach

The numerical modeling accuracy was examined by comparing the simulated results against published impact and blast tests in the open literature. Two experimental tests were used to validate the bare RC pier modeling approaches, including a drop hammer impact test on RC beams and a reduced-scale blast test of an RC column from a building frame [24,31]. The simulated results matched reasonably well with the tested results. The validation results were provided in the previous studies by the authors [24,31]. For the retrofitted RC piers, validation of selected modeling techniques involved comparing predictions against results from two separate experimental studies of CFRP-wrapped RC structural components subjected to impact and blast loads. Validation studies details are provided elsewhere [24]. The studies showed that developed models were able to provide reasonable predictions of damage patterns and dynamic behavior of the bare and retrofitted RC structural components subject to an impact and blast.

## 3. Numerical Studies

### 3.1. Model Description

**Figure 2:**
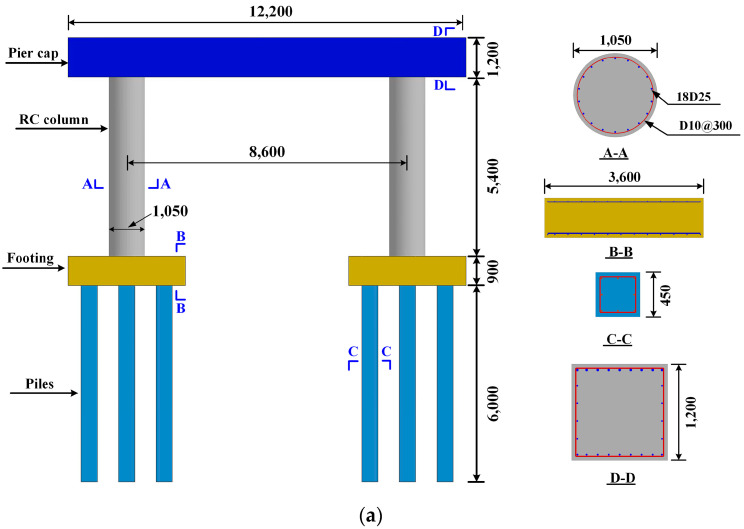

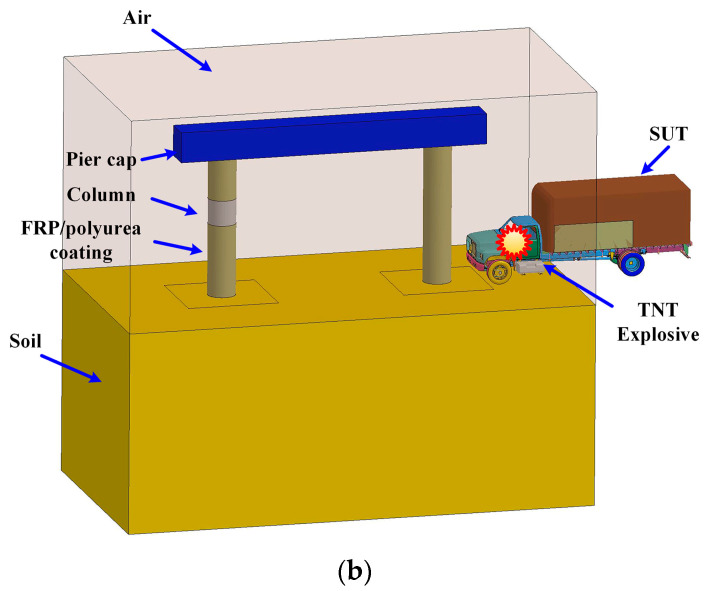
Pier details and numerical model with retrofit schemes: (**a**) pier geometry (unit: mm); (**b**) numerical model.

A representative dual-column pier taken from an FHWA design example was modified and subjected to a combined medium-size vehicle collision and air blast. The pier was then strengthened with CFRP wrap or polyurea coating to examine the pier blast and impact performance. The dual-column pier consisted of two circular columns, pile foundations, and a cap at the column top, as shown in Figure 2. The circular columns had a diameter of 1050 mm and a height of 5400 mm. The columns were reinforced with 18 No. 25 longitudinal bars and No. 10 hoops spaced 300 mm along the height, with all designs completed following the AASHTO Bridge Design Manual. The columns were supported by a pile foundation system involving a footing with dimensions of 3600 mm × 3600 mm × 900 mm and sitting on eight 450 mm wide square piles. The length of each pile was 6000 mm. Columns were connected to a rectangular cap that was 1200 mm deep. In situ retrofits were coated to the full height of both columns. The CFRP had a thickness of 3 mm for the simulations, and the 9 mm thick polyurea was used to strengthen both columns. The polyurea thickness was selected from the preliminary simulations, in which the 9 mm thick polyurea-coated column had a similar axial capacity to the 3 mm thick FRP-wrapped column.

The superstructure dead load was modeled as an axial preload at the cap top and was 6% of the column’s nominal axial capacity on each column. The axial load increased from zero to the designed value for a period of 0.03 s before the truck collision. Then, the SUT impacted the pier at velocities (*v*_0_) between 65 km/h and 120 km/h, which covered the range of highway speed limits. After the collision, an air blast was activated in the simulation at a scale distance (Z) between 0.20 m/kg^1/3^ and 0.30 m/kg^1/3^. This range was determined from the NCHRP Report 645 design rules [32], in which the blast performance of the bridge column is suggested to be examined for Z ≤ 0.6 m/kg^1/3^. To quantify the damage degrees and examine the damage status for the bridge piers during the multi-hazard events, a damage index ($\lambda_{DI}$) based on the residual column lateral deflection was used in this study and calculated using $\lambda_{DI}=d_{r}/D_{c}$, where $d_{r}$ is the residual lateral deflection and $D_{c}$ is the column diameter. As suggested in the studies for RC structure impact behaviors [33], the damage level can be identified through the damage index: 0 ≤ $\lambda_{DI}$ < 0.03 signifies minor damage, 0.03 ≤ $\lambda_{DI}$ < 0.1 signifies moderate damage, and $\lambda_{DI}$ ≥ 0.1 signifies severe damage.

### 3.2. Simulation Results

#### 3.2.1. Pier Damage Accumulation

**Figure 3:**
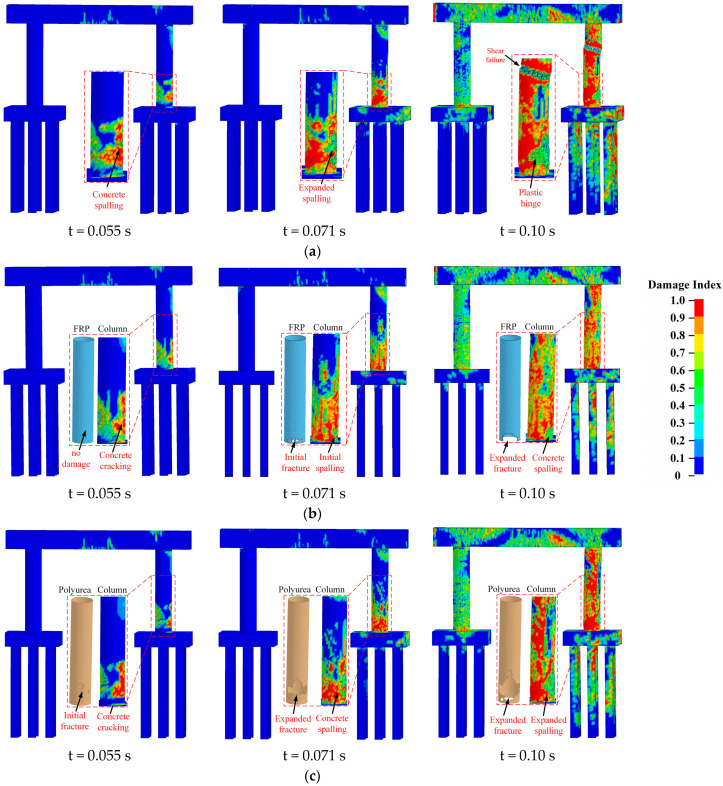
Damage statuses and propagations: (**a**) bare pier; (**b**) CFRP-retrofitted pier; (**c**) polyurea-retrofitted pier.

**Figure 4:**
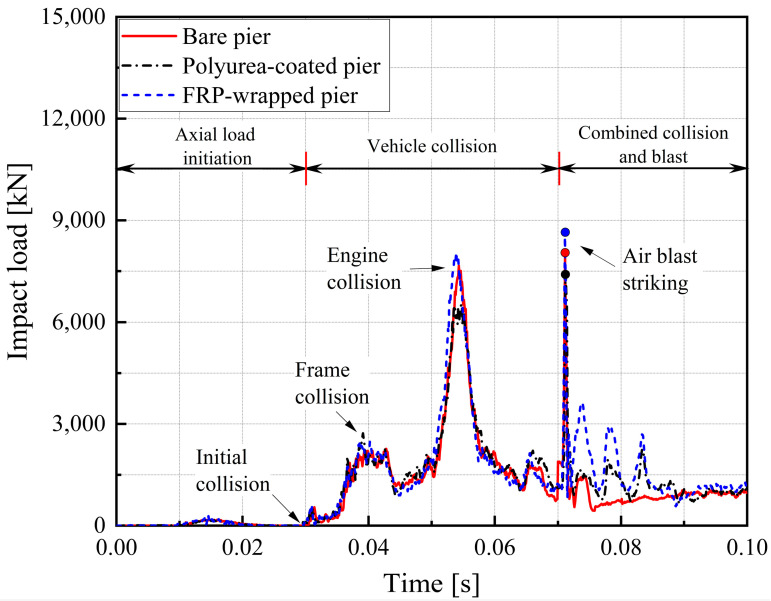
Impact load time histories for bare and retrofitted piers.

Figure 3 details the representative concrete and CFRP/polyurea erosion damage propagation during the multi-hazard events. To clearly represent the pier response to the combined collision and blast loads, the impact forces for bare and retrofitted piers were analyzed, as shown in Figure 4, and classified into three phases. In the initial phase (0–0.03 s), the axial load and gravity were gradually applied to the bridge piers with no interactions between the piers and the truck. The SUT initially impacted the piers at t = 0.03 s to promote the increased impact loads at the collision region. At t = 0.04 s, the first load peak was obtained due to the SUT frame collision, with slight variations for three bridge piers. Initial concrete cracking was observed at the impacted column for the bare pier, while the FRP and polyurea retrofitted pier were still intact. At t = 0.055 s, the vehicle engine impacted the bridge column to generate a second load peak. The peak loads on the polyurea retrofitted column were lower due to crash buffer functions from the elastic properties of polyurea. For the bare pier, the concrete began spalling in the collision region, while cracking occurred on the impacted side of the column for the FRP and polyurea retrofitted piers. Initial fracturing of the polyurea was also observed.

As the SUT impact loads decreased and stabilized, an air blast was activated at t = 0.07 s, resulting in a third load peak. For the bare pier, a 45-degree shear crack formed in the collision region and propagated to the non-impacted side at the base, along with severed concrete spalling. For the CFRP-retrofitted pier, concrete began spalling at the impact point with the extended cracks to the non-impacted side of the column, and an initial fracture of CFRP wrap occurred on the impacted side at the base. For the polyurea-coated pier, concrete was spalled on the front side with the propagation of cracks at the base, and the polyurea fracturing was expanded in the collision region. In the post-peak phase, shear failure occurred in the mid-height region of the impacted column for the bare pier, with the development of a plastic hinge at the base, as shown in Figure 3a. A high risk of collapse existed for the bare pier due to the shear failure of the impacted column. For the CFRP retrofitted pier, concrete spalling was created at the base, and cracks propagated through the non-impact side and the top of the column, as shown in Figure 3b. For the polyurea-coated pier, concrete spalling expanded on the front side of the impacted column with the development of 45-degree shear cracks at the top and base, as shown in Figure 3c. After retrofitting the columns, the damaged piers performed adequately under operational conditions. As a result, the demands for the bare and retrofitted bridge piers in the multi-hazard events were controlled by two impact load peaks, including the SUT engine collision and airwave striking on the impacted columns. Additionally, using the CFRP wrap and polyurea coating effectively enhanced the bridge pier’s resistance against the multi-hazard demands and improved the pier’s performance under the combined collision–blast loading.

#### 3.2.2. Pier Dynamic Behaviors

**Figure 5:**
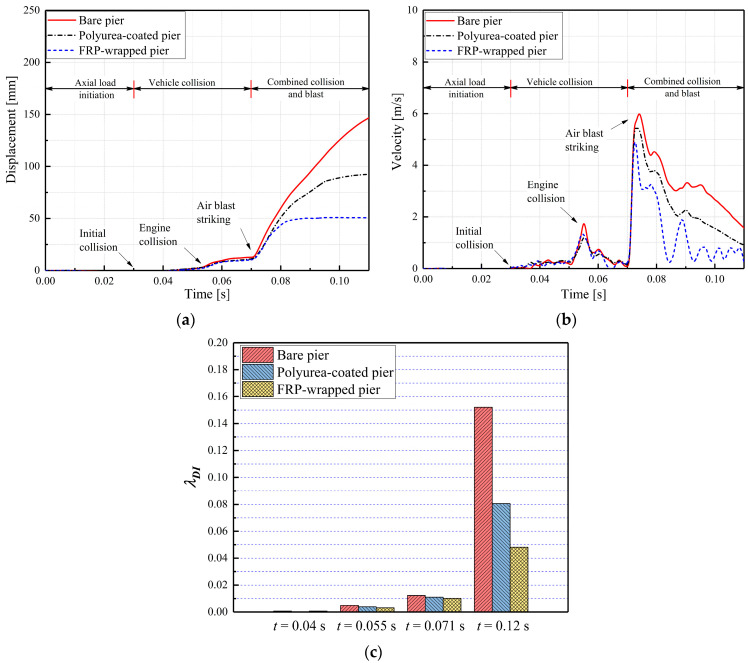
Dynamic behaviors of bare and retrofitted piers: (**a**) column displacement; (**b**) column velocity; (**c**) damage indices.

Figure 5 shows the dynamic behaviors of the bare and retrofitted piers during the multi-hazard events. As shown in Figure 5, column displacements and velocities were small before the engine collision, which was attributed to the crushable characteristics and deformation of the truck’s frontal frame dissipating energy. The engine collision produced a rapid increase in displacement near the impact region, along with the first column velocity peak. During the SUT truck collision phase, the displacement of the bare pier was about 33% higher than the CFRP-wrapped pier and 20% larger than the polyurea-coated pier. At the engine collision (t = 0.055 s), the damage indexes for the CFRP-wrapped and polyurea-coated piers were 0.0031 and 0.0038, respectively, which represented practically identical damage levels for the retrofitted piers. The CFRP wrap effectiveness was similar to the polyurea effectiveness in mitigating the truck collision and improving the pier collision resistance. When the air blast was activated in the events, the column displacement and velocity rapidly increased due to accumulated pier damage. In the post-peak phase, the bare pier’s velocity was much higher than those obtained for the CFRP-wrapped and polyurea-coated piers, which indicated that the CFRP and polyurea dissipated energy to protect the bridge piers. Additionally, the CFRP wrap was able to dissipate more energy compared with the polyurea coating. The final displacement of the dual-column pier was reduced by 68.3% and 41% using the CFRP wrap and polyurea coating, respectively. The damage indexes decreased from 0.15 to 0.05 and 0.08 for the CFRP- and polyurea-retrofitted piers, respectively. These indices reflected a transition from severe damage to moderate damage. Thus, the CFRP wrap and polyurea coating effectively reduced the pier displacement and absorbed the energy from the combined collision–blast loads.

### 3.3. Parametric Sensitivity Analysis

Parametric analyses were conducted in this study to examine the influence of demand and design parameters on the effectiveness of studied retrofit schemes. The analyses were intended to enable the identification of controlling parameters to optimize each retrofit scheme for bridge pier performance improvement.

#### 3.3.1. Effect of the Truck Velocity

**Figure 6:**
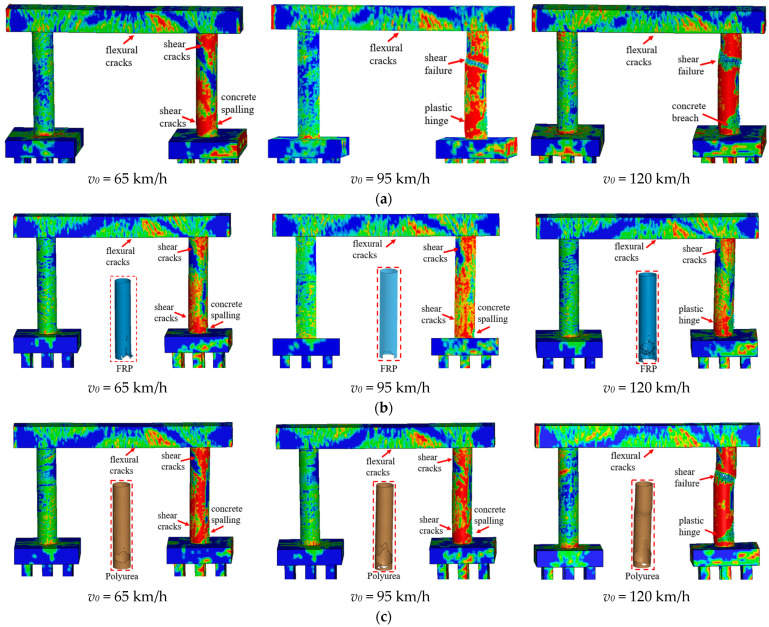
Failure modes of bare and retrofitted piers at various velocities (*Z* = 0.25 m/kg^1/3^): (**a**) bare pier; (**b**) CFRP-retrofitted pier; (**c**) polyurea-retrofitted pier.

Figure 6 compares the failure modes for bare and retrofitted piers for varying SUT impact velocities. It can be seen from Figure 6 that the pier performance improved using CFRP wrap and polyurea coating with less concrete erosion at the mid-height and the base of impacted columns. For the case with *v*_0_ = 65 km/h and *Z* = 0.25 m/kg^1/3^, concrete spalling and shear cracks occurred at the base of the impacted columns for both the bare and retrofitted piers. All piers were deemed operational. For the case with *v*_0_ = 95 km/h and Z = 0.25 m/kg^1/3^, the combined loads resulted in shear failure at the mid-height and a plastic hinge at the base of impacted columns for the bare pier, which had severe damage and an unsafe condition. Using the CFRP and polyurea, the impacted columns experienced concrete spalling at the base, and the piers were operational with repairable damage. As the truck velocity increased to 120 km/h combined with *Z* = 0.25 m/kg^1/3^, shear failure occurred at the mid-height, along with a concrete breach at the base in the impacted column for the bare pier. For the CFRP-wrapped pier, the impacted column sustained a plastic hinge at the base, and the pier could remain in operation. For the polyurea-coated pier, the impacted column failed in shear at the mid-height, and a plastic hinge was formed at the base.

**Figure 7:**
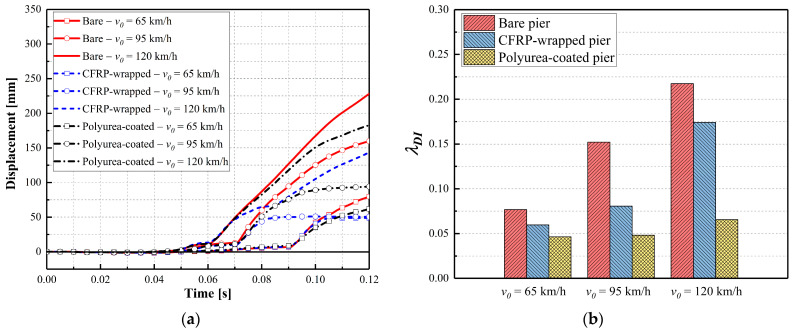
Dynamic responses of bare and retrofitted piers at various velocities (*Z* = 0.25 m/kg^1/3^): (**a**) column displacement; (**b**) damage index.

Figure 7 illustrates pier displacement and associated damage indices for varying velocities. As shown in Figure 7a, the CFRP-wrapped pier displacements were approximately 59% less when compared with the bare pier, and the polyurea coating reduced the final displacements by approximately 30%. These maximum polyurea-coated pier displacements were approximately 20% larger than the displacements experienced by the CFRP-wrapped piers under similar demands. For the case with *v*_0_ = 65 km/h, performance improvements were less pronounced than the other velocities with a smaller reduction in displacement. The damage indices for the CFRP-wrapped piers ranged between 0.046 and 0.065, which was lower than 0.1 and represented moderate damage for the piers, as shown in Figure 7b. The damage indices for the polyurea-coated piers were higher than for the CFRP-wrapped piers. The damage index was larger than 0.1 for *v*_0_ = 120 km/h, indicating severe pier damage and ineffective retrofits for the dual-column pier system. Therefore, both in situ retrofit schemes could enhance the multi-hazard resistance and improve the performance of the bridge pier under the combined collision and blast. For the parameters examined for this study, the CFRP wrap was preferred for truck velocities of *v*_0_ = 120 km/h.

#### 3.3.2. Effect of the CFRP Strength

**Figure 8:**
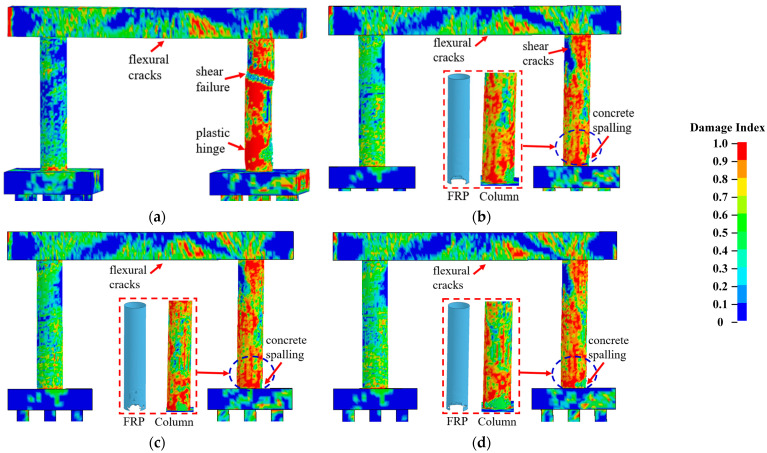
Failure modes of CFRP-wrapped piers with varied strengths: (**a**) bare pier; (**b**) *f_FRP_* = 1095 MPa; (**c**) *f_FRP_* = 1950 MPa; (**d**) *f_FRP_* = 2280 MPa.

**Figure 9:**
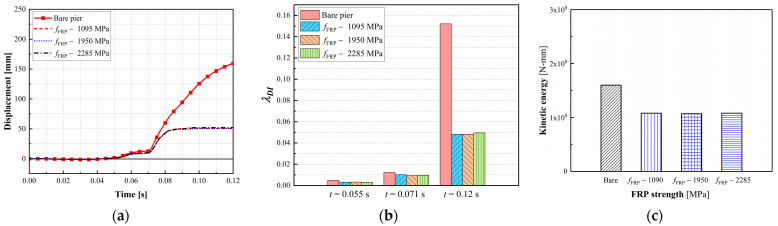
Dynamic responses of CFRP-wrapped piers with varied strengths: (**a**) column displacement; (**b**) column velocity; (**c**) damage indexes.

Three CFRP strengths, which were selected from available test data from previous studies in the open literature [5,6], were examined in this study to improve the bridge piers. Figure 8 compares the failure modes of the bridge piers retrofitted with three CFRP strengths. With different CFRP strengths, the bridge piers exhibited similar damage, including concrete spalling at the base and concrete cracks developed through the impacted column. These piers were deemed operational, and the damaged column could be repaired under operations. Figure 9a,b plot the column displacement and associated damage indices at the critical time. As shown in Figure 9, the variation in the CFRP strength resulted in little changes in the pier displacement during the multi-hazard events, and all damage indices at the final state approximated to 0.05 for all retrofitted piers. Additionally, the energy dissipation was the same for these CFRP-retrofitted piers, with an insignificant correlation between the energy dissipation rate and the CFRP strengths. The performance of the CFRP-retrofitted dual-column pier did not vary as a function of the CFRP strength. 

#### 3.3.3. Effect of the CFRP Thickness

**Figure 10:**
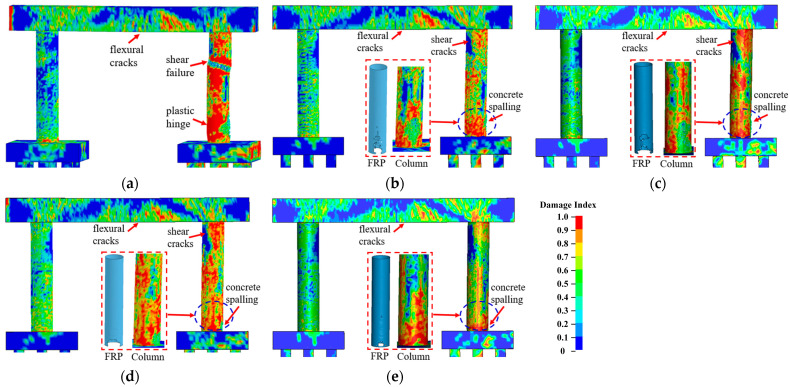
Failure modes of CFRP-wrapped piers with varied thicknesses: (**a**) bare pier; (**b**) *t_FRP_* = 1 mm; (**c**) *t_FRP_* = 2 mm; (**d**) *t_FRP_* = 3 mm; (**e**) *t_FRP_* = 6 mm.

Numerical simulations were conducted with varied CFRP thicknesses (i.e., 1 mm, 2 mm, 3 mm, and 6 mm) to assess the performance of a dual-column pier against multi-hazard demands. Figure 10 details the failure modes of CFRP-wrapped piers with varied CFRP thicknesses. It can be seen from Figure 10 that the use of the CFRP wrap through the entire columns significantly mitigated the pier damage and prevented shear failure at the mid-height of impacted columns. As the CFRP thickened, the damage intensity was reduced, with decreased spalled concrete in the collision region. For the bare pier, shear failure occurred at the mid-height of the impacted column, along with a plastic hinge at the base, which was considered unsafe to initiate collapse. The 1 mm and 2 mm thick CFRP wrap resulted in concrete spalling at the base of impacted columns, which was repairable under operational conditions. When the CFRP thickness was larger than 3 mm, minor concrete spalling was observed at the base of the impacted column.

**Figure 11:**
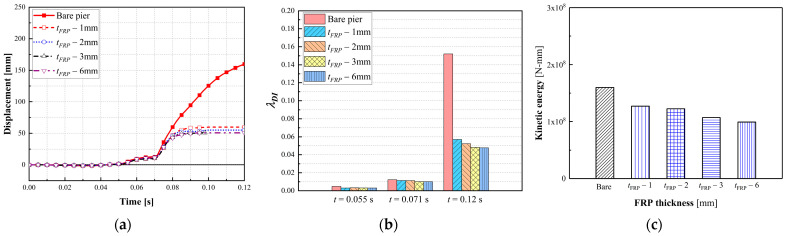
Dynamic responses of CFRP-wrapped piers with varied thicknesses: (**a**) column displacement; (**b**) column velocity; (**c**) damage indices.

Figure 11a,b show column displacement and associated damage indices. When the 1 mm thick CFRP was wrapped, the maximum pier displacement was significantly reduced by 60% compared with the bare pier. The column displacement decreased as the CFRP thickness increased. The 3 mm and 6 mm thick CFRP wrap produced a similar pier displacement of 50.6 mm. Additionally, the damage index reduced from 0.15 to 0.057 when the 1 mm thick CFRP was utilized, indicating a transition from severe damage to moderate damage. The damage indices for the CFRP-retrofitted piers with varied thicknesses were in the range of 0.056–0.047. Increased CFRP thickness reduced the column kinetic energy, identifying the improvement in energy dissipation, as shown in Figure 11c. However, a clear limit to the CFRP wrap thickness existed. When the FRP increased from 3 mm to 6 mm, the effectiveness of the CFRP wrap was not prominent, with similar displacement and dissipated energy. This was due to the CFRP thickness of 3 mm being sufficient to mitigate the collision and blast effects on the pier’s performance. The CFRP with a thickness of 3 mm possessed adequate strength to resist some fiber layer damage produced by a vehicle collision and continued providing confinement on the bridge columns.

#### 3.3.4. Effect of the Polyurea Thickness

**Figure 12:**
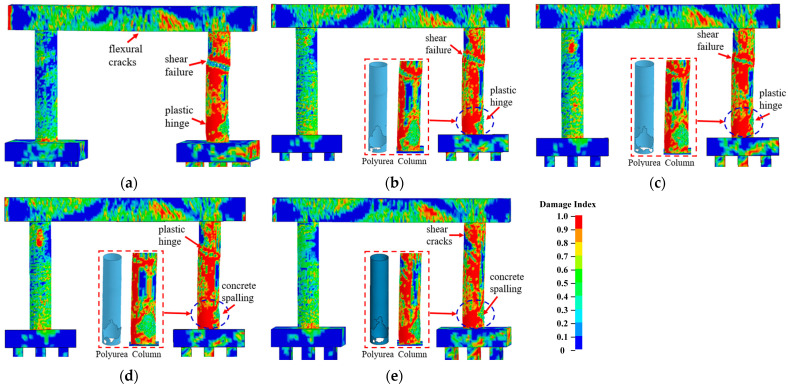
Failure modes of polyurea-coated piers with varied thicknesses: (**a**) bare pier; (**b**) *t_Pol_* = 6 mm; (**c**) *t_Pol_* = 7 mm; (**d**) *t_Pol_* = 8 mm; (**e**) *t_Pol_* = 9 mm.

Dynamic behaviors of piers coated with 6 mm, 7 mm, 8 mm, and 9 mm thick polyurea were examined to evaluate the effect of the polyurea thickness on the dual-column pier’s performance. Figure 12 shows the failure modes of polyurea-coated piers with varied polyurea thicknesses. As shown in Figure 12, an increased polyurea thickness reduced the damage levels. Shear failure was observed at the mid-height of the impacted column retrofitted using 6 mm thick polyurea, which created an unsafe condition for the bridge pier. An increase in thickness from 6 mm to 8 mm led to the development of a plastic hinge rather than shear failure. As the thickness increased to 8 mm and 9 mm, the combined collision–blast loads produced concrete spalling in the collision region of the impacted column, which was repairable under operational conditions.

**Figure 13:**
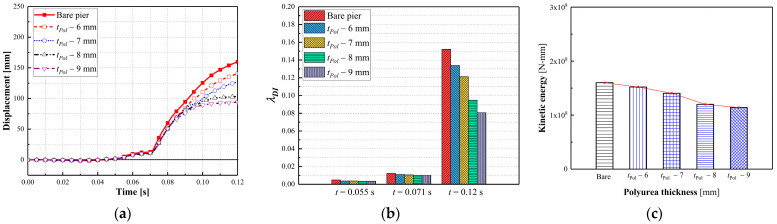
Dynamic responses of polyurea-coated piers with varied thicknesses: (**a**) column displacement; (**b**) column velocity; (**c**) damage indices.

Figure 13a,b plot the column displacement and associated damage indices during the events. The column displacements reduced with an increase in the polyurea thickness, and at a polyurea thickness of 9 mm, the maximum column displacement was 40% less than that experienced by a bare pier. An increase in polyurea thickness from 6 mm to 9 mm led to a significant decrease in the damage index from 0.13 to 0.80, which represented a transition from severe damage to moderate damage. Additionally, the pier kinetic energy reduced as the polyurea thickness increased, identifying the enhanced polyurea energy dissipation, as shown in Figure 13c. As the polyurea thickness increased from 7 mm to 8 mm, the pronounced decrease in the damage index and kinetic energy demonstrated the prominent effectiveness of the polyurea in mitigating the effects of the collision and blast and improving the pier’s performance. The polyurea thickness of 9 mm was sufficient to increase the pier’s resistance against the combined collision–blast loads and prevent a potential collapse after the multi-hazard event.

#### 3.3.5. Effect of the In Situ Retrofit Location

**Figure 14:**
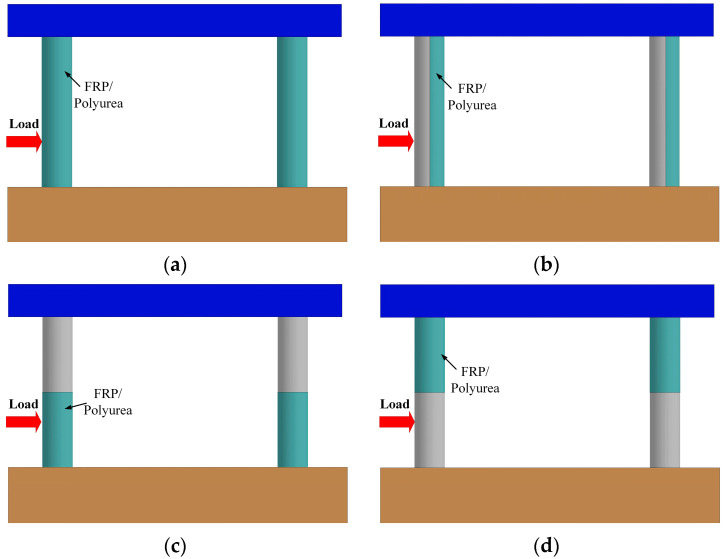
In situ retrofit locations for bridge piers: (**a**) full retrofit; (**b**) half retrofit on the non-impact side; (**c**) half retrofit at the column’s base; (**d**) half retrofit at the column’s top.

To investigate different retrofit schemes and analyze their retrofit effectiveness, it was of interest to examine whether the optimized placement of the wrap onto the column offered any benefit with respect to multi-hazard resistance. Three additional locations were selected to enhance the shear capacity at critical locations identified from representative failure modes of bare piers. The locations consisted of coating half the circumference on the non-impact side of all columns, coating half the height at the base of all columns, and coating half the height at the top of all columns, as illustrated in Figure 14.

**Figure 15:**
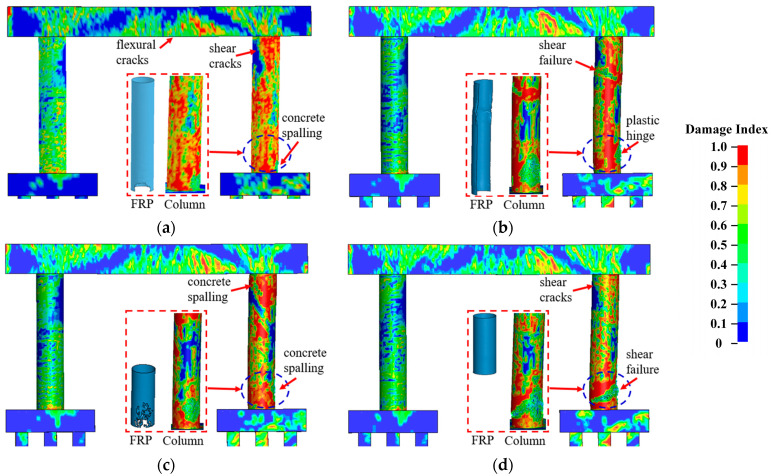
Pier damage for four CFRP wrapping schemes: (**a**) full wrap; (**b**) half wrap on the non-impact side; (**c**) half wrap at the column’s base; (**d**) half wrap at the column’s top.

**Figure 16:**
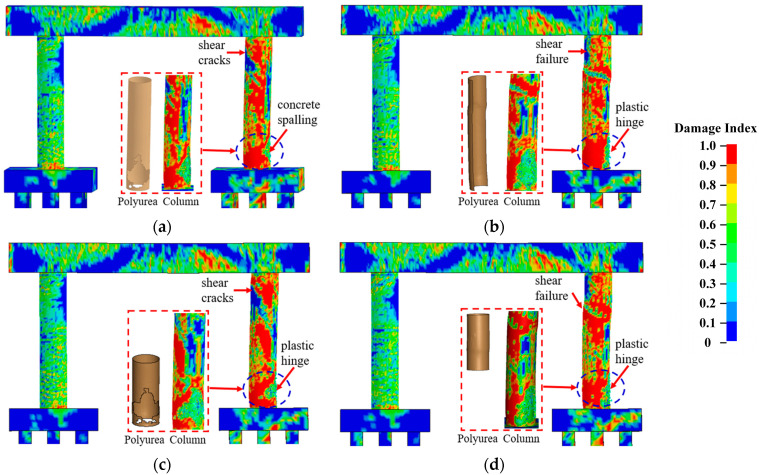
Pier damage for four polyurea coating schemes: (**a**) full coating; (**b**) half coating on the non-impact side; (**c**) half coating at the column’s base; (**d**) half coating at the column’s top.

Figure 15 and Figure 16 detail the failure modes of the CFRP- and polyurea-retrofitted piers, respectively. In the cases with full coating, the piers sustained concrete spalling at the base of the impacted columns, which were reparable under operational conditions. When half the circumference on the non-impact side of all columns was coated, the impacted columns experienced shear failure at the mid-height and a plastic hinge at the base, which was considered unsafe for the bridge piers. In the cases with coating half the height at the base of all columns, concrete spalling was observed in the CFRP-wrapped pier, and a plastic hinge was developed in the impacted column of the polyurea-coated pier. These piers could remain in operation, but the damaged column would need repair. When the column top was retrofitted with CFRP and polyurea down to half the height, shear failure occurred to the impacted column, identifying the ineffective improvement in pier resistance against multi-hazard demands.

**Figure 17:**
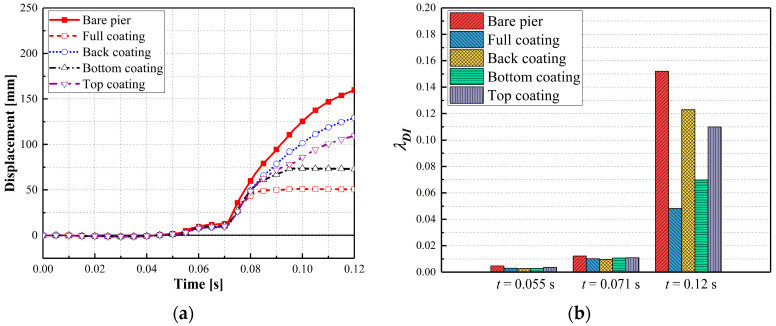
Dynamic responses for four CFRP wrapping schemes: (**a**) column displacement; (**b**) damage indices.

**Figure 18:**
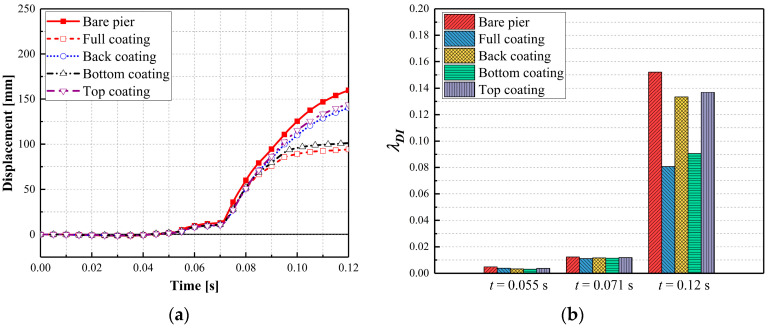
Dynamic responses for four polyurea coating schemes: (**a**) column displacement; (**b**) damage indices.

Figure 17 and Figure 18 plot the pier displacement and associated damage index during the multi-hazard events, in which the results matched the damage patterns and failure modes observed in Figure 15 and Figure 16. For the full wrap and half wrap at the column base, the column displacements were less than those experienced by the piers with other schemes. The damage indices for the piers with half coating on the non-impact side and at the column top were higher than 0.1, identifying severe damage. For the piers with a full coating and half coating at the column base, the damage indices ranged between 0.48 and 0.86, representing moderate damage for the piers, which could remain in operation. Thus, retrofitting half the column height at the base was an optimal approach that could enhance the dual-column pier’s resistance against extreme demands and achieve an economic design.

## 4. Summary and Conclusions

This study numerically investigated the behavior of dual-column bridge piers retrofitted using CFRP and polyurea under the combined medium-size truck collision and air blast. Numerical simulations were conducted to examine the dynamic behaviors of the bare and retrofitted dual-column piers to evaluate the retrofit effectiveness. Parametric studies were performed to explore optimal retrofit schemes for bridge pier multi-hazard resistance enhancement. Results from the studies indicated the following:

(1) Coating the entire height of all pier columns could effectively mitigate the effects of the combined collision and blast. Retrofitting half of all columns at the base offered a similar performance when the collision was aligned with the pier’s long axis.

(2) The CRFP thickness influenced the behavior, with a thicker wrap reducing the damage caused by the collision and blast. A limit to the effectiveness was observed, however, with similar column damage, displacement, and kinetic energy occurring as the CFRP thickness increased from 3 mm to 6 mm. The CFRP strength had a minor effect on the retrofit effectiveness.

(3) For the variables and demands that were examined, an increased polyurea thickness also affected the pier’s performance. The most dramatic effects occurred when the polyurea thickness increased between 7 mm and 8 mm, as failure was mitigated.

(4) For the multi-column piers examined in this study, the effectiveness of the CFRP wrap with a 3 mm thickness was close to the polyurea effectiveness with a 9 mm thickness for mitigating the collision and blast effects on the pier performance to ensure the pier integrity. In multi-hazard events with a truck velocity of higher than 120 km/h, the CFRP wrap was identified as the optimal retrofit scheme.

(5) To further investigate in situ retrofit schemes for highway bridges under the combined collision–blast loading and improve the safety of bridge components and systems, future work is needed: (i) develop a performance-based method to use the efficient in situ retrofit scheme based on the construction needs and resource availability, (ii) investigate the performance of the entire bridge system strengthened using the CFRP wrap and polyurea coating in the multi-hazard events with vehicle collisions and blasts, and (iii) conduct full-scale testing on bridge columns retrofitted with CFRP and polyurea when subjected to a combined vehicle collision and air blast to validate the simulated results.

## Figures and Tables

**Table 1:** Material parameters.

Material	Parameters			
Concrete	Mass density	Compressive strength	Maximum aggregate size	
	2380 kg/m^3^	28 MPa	19 mm	
Steel	Mass density	Elasticity modulus	Poisson’s ratio	Yield stress
	7830 kg/m^3^	2 × 10^5^ MPa	0.3	475 MPa

**Table 2:** Soil material parameters.

Parameter	Specific Gravity	Bulk Modulus	Shear Modulus	Friction Angle	Cohesion
Value	2.65	146 MPa	56 MPa	35°	5 × 10^−6^ MPa

**Table 3:** CFRP material parameters.

Parameter	Longitudinal Modulus	Transverse Modulus	In-Plane Shear Modulus	Out-of-Plane Shear Modulus	Poisson’s Ratio
Value	118 GPa	5.5 GPa	4.8 GPa	4.8 GPa	0.0127
Parameter	Longitudinal Tensile Strength	Transverse Tensile Strength	Longitudinal Compressive Strength	Transverse Compressive Strength	In-Plane Shear Strength
Value	712.9 MPa	1095 MPa	26.4 MPa	84.4 MPa	84.3 MPa

## Data Availability

Data is available on request due to restrictions. The data presented in this study are available on request from the corresponding author. The data are not publicly available because this study is part of an ongoing large research project.
